# Long-Term Cardiovascular Risk and Maternal History of Pre-Eclampsia

**DOI:** 10.3390/jcm14093121

**Published:** 2025-04-30

**Authors:** Pasquale Palmiero, Pierpaolo Caretto, Marco Matteo Ciccone, Maria Maiello, on behalf of the I.C.I.S.C.U. (Italian Chapter of International Society Cardiovascular Ultrasound)

**Affiliations:** 1 ASL Brindisi, Cardiology Equipe, District of Brindisi, 72100 Brindisi, Italy; mariamaiello@iciscu.org; 2 Medical School, University of Bari, 70122 Bari, Italy; 3 University Cardiology Unit, Interdisciplinary Department of Medicine, Polyclinic University Hospital, 70124 Bari, Italy; pierpaolo.caretto@uniba.it (P.C.); marcomatteo.ciccone@uniba.it (M.M.C.)

**Keywords:** pre-eclampsia, hypertension, cardiovascular disease, maternal morbidity

## Abstract

Pre-eclampsia is a severe pregnancy complication affecting 5–8% of pregnancies worldwide, marked by high blood pressure and organ damage typically occurring after 20 weeks of gestation. It is a leading cause of maternal and fetal morbidity and mortality. Though its exact cause is unknown, it involves placental abnormalities and improper blood vessel development. Risk factors include a history of pre-eclampsia, chronic hypertension, diabetes, obesity, and autoimmune disorders. Symptoms include high blood pressure, proteinuria, headaches, vision changes, and abdominal pain. Untreated, it can lead to seizures, stroke, preterm birth, or death. Delivery is the definitive treatment, with management strategies such as monitoring and blood pressure control. Pre-eclampsia significantly increases long-term cardiovascular disease (CVD) risks, including hypertension, ischemic heart disease, and stroke, linked to shared mechanisms like endothelial dysfunction and inflammation. Women with severe or recurrent pre-eclampsia have heightened risks, often developing chronic hypertension within a decade postpartum. It also impacts offspring, with daughters at elevated risk for pre-eclampsia and CVD. Hypertensive disorders of pregnancy, including pre-eclampsia, induce changes like left ventricular hypertrophy and diastolic dysfunction, raising risks for heart failure with preserved ejection fraction and coronary atherosclerosis. Overlapping with peripartum cardiomyopathy, pre-eclampsia underscores a spectrum of pregnancy-related cardiovascular disorders. Long-term monitoring and lifestyle interventions are crucial for managing risks, with research into genetic and biological mechanisms offering the potential for targeted prevention.

## 1. Introduction

**Figure 1:**
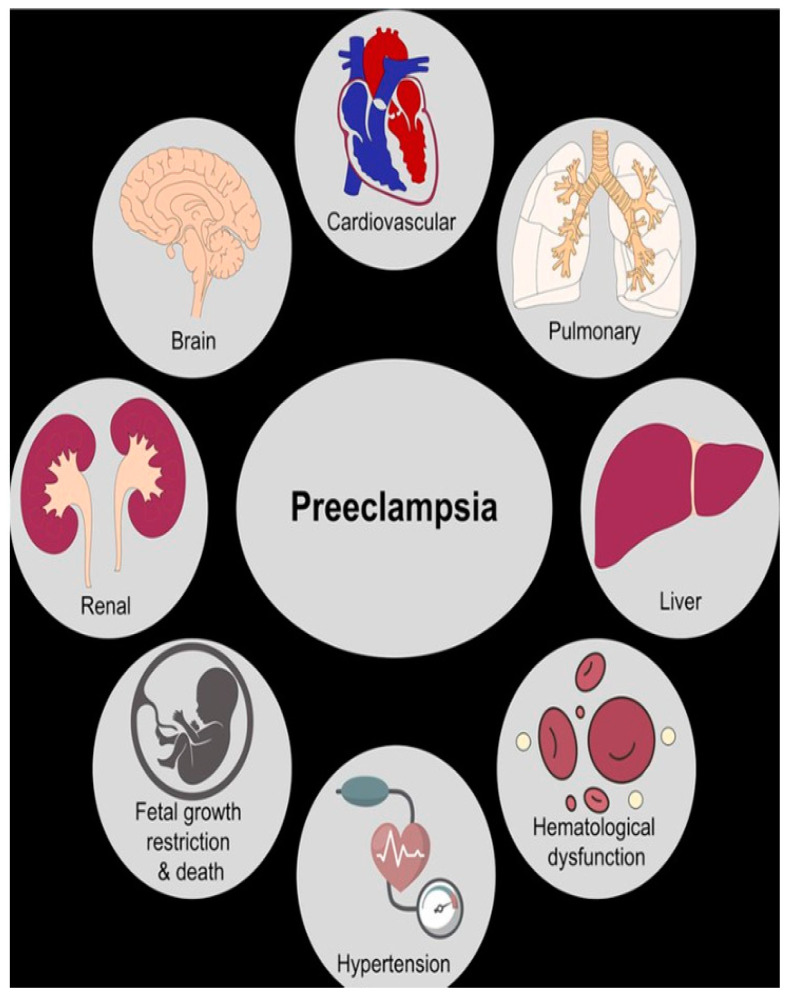
Pre-eclampsia damages numerous organs and systems.

Pre-eclampsia is a pregnancy-related condition characterized by high blood pressure (hypertension) and damage to many organs and systems, often kidneys or liver, Figure 1. It typically occurs after 20 weeks of gestation and can also develop postpartum. Pre-eclampsia affects approximately 5–8% of pregnancies worldwide and is a leading cause of maternal and fetal morbidity and mortality.

The exact cause of pre-eclampsia is not fully understood, but it is believed to involve abnormalities in the placenta and improper development of blood vessels during early pregnancy. Risk factors include a history of pre-eclampsia, first-time pregnancy, multiple gestation (e.g., twins), chronic hypertension, diabetes, obesity, metabolic syndrome, and autoimmune disorders. Pre-eclampsia is increasingly seen not only as a pregnancy complication but also as a significant predictor of cardiovascular risk later in life. This review explores the long-term cardiovascular risks associated with pre-eclampsia and examines the implications of maternal history in understanding and mitigating these risks.

## 2. Cardiovascular Risk in Women with a History of Pre-Eclampsia

**Figure 2:**
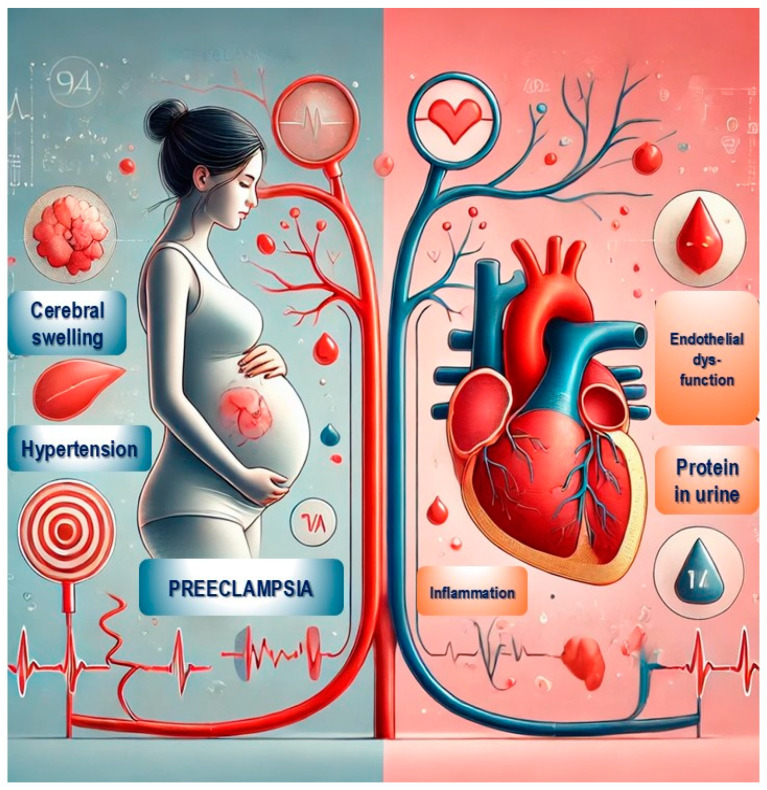
Cardiovascular risk in women with a history of pre-eclampsia.

Studies consistently show that women who experience pre-eclampsia are at increased risk for cardiovascular diseases later in life, including hypertension, ischemic heart disease, stroke, and heart failure, Figure 2. Research has highlighted that this increased risk is not merely a residual effect of pregnancy but is instead linked to the shared pathophysiological mechanisms underlying both pre-eclampsia and cardiovascular conditions, such as endothelial dysfunction, inflammation, and metabolic irregularities [1]. Women with severe or recurrent pre-eclampsia or those who delivered preterm have an even greater risk, with some evidence suggesting up to a twofold increase in cardiovascular disease compared to women who experienced uncomplicated pregnancies [2].

**Figure 3:**
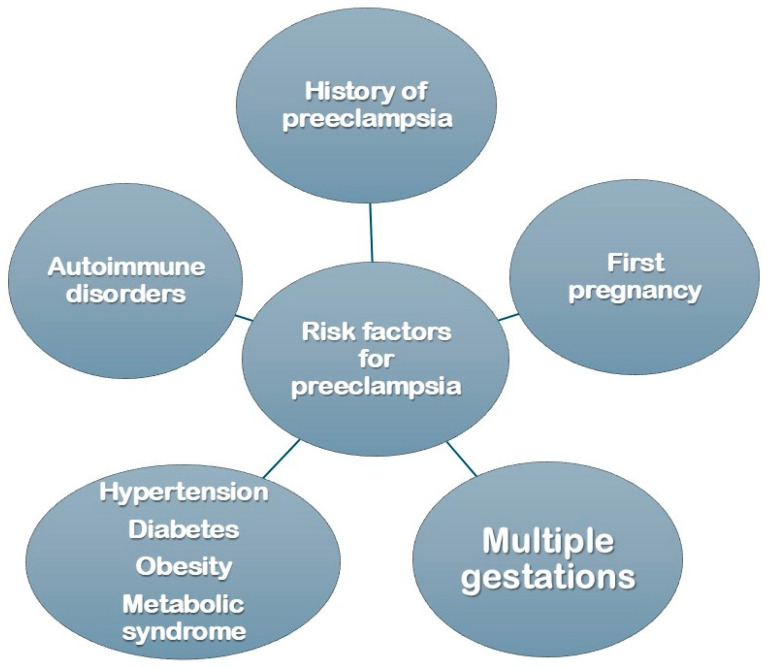
Risk factors for pre-eclampsia.

Furthermore, pre-eclampsia often precedes other cardiovascular risk factors like hypertension, diabetes, and obesity, which contribute to a heightened risk profile for these women as they age. Women with pre-eclampsia are at a significantly increased risk of developing chronic hypertension within 10 years postpartum [3]. Additionally, the history of pre-eclampsia is increasingly being recognized as a critical risk factor in cardiovascular assessments. The American Heart Association now includes pre-eclampsia in its guidelines for cardiovascular risk evaluation, highlighting the importance of monitoring blood pressure, cholesterol levels, and lifestyle factors in women with this history. A visual representation of the range of risk factors for pre-eclampsia is shown in Figure 3 [4]. Early intervention strategies, such as lifestyle modifications and regular monitoring, are recommended to help mitigate these long-term risks.

## 3. Maternal History and Genetic Implications

Maternal history of pre-eclampsia also has implications for offspring, suggesting that genetic or familial factors may play a role. Daughters of women who experienced pre-eclampsia are at an elevated risk of pre-eclampsia in their pregnancies, and studies indicate they may also inherit a heightened susceptibility to cardiovascular diseases (CVD) [5].

## 4. Hypertensive Disorders of Pregnancy (HDP)

Hypertensive disorders of pregnancy (HDP) encompass a spectrum of conditions characterized by elevated blood pressure during pregnancy. These disorders, which include chronic hypertension, gestational hypertension, pre-eclampsia [6], and chronic hypertension with superimposed pre-eclampsia, are among the leading causes of maternal and perinatal morbidity and mortality worldwide. Chronic hypertension is diagnosed before pregnancy or within the first 20 weeks of gestation and often persists postpartum. Women with chronic hypertension are at increased risk of developing pre-eclampsia later in pregnancy. Gestational hypertension occurs after 20 weeks of gestation without proteinuria or other systemic involvement. While it is typically mild, it requires monitoring as it can progress to pre-eclampsia. Pre-eclampsia, a severe form of HDP, is defined by hypertension and signs of organ damage, such as proteinuria, liver dysfunction, or thrombocytopenia. It poses serious risks, including placental abruption, eclampsia (seizures), and HELLP syndrome (hemolysis, elevated liver enzymes, and low platelets). Management of HDP depends on the severity of the condition and gestational age. For chronic and gestational hypertension, treatment includes antihypertensive medications, lifestyle changes, and close monitoring. Severe pre-eclampsia often necessitates hospitalization and early delivery to prevent complications. Complications of untreated HDP can affect both mother and fetus, resulting in preterm delivery, fetal growth restriction, maternal organ failure, and long-term cardiovascular risks. Postpartum follow-up is critical to manage persistent hypertension and evaluate future risks.

## 5. Pre-Eclampsia and the Development of Arterial Hypertension

Pre-eclampsia has been identified as a major risk factor for chronic hypertension in women post-pregnancy. Studies have shown that about 50% of women with a history of pre-eclampsia are two to four times more likely to develop chronic hypertension within five to fifteen years postpartum [3]. In those with normotensive pregnancies, this risk is heightened in cases of early-onset or severe pre-eclampsia, with research indicating that blood pressure abnormalities may persist or develop years after pregnancy [7]. Arterial hypertension resulting from pre-eclampsia is linked not only to the pregnancy period itself but also to underlying mechanisms, including endothelial dysfunction [8], oxidative stress [9], and inflammation, renin-angiotensin-aldosterone system dysregulation [10], and genetic and epigenetic factors that continue to impact cardiovascular health beyond pregnancy. Recognizing arterial hypertension is important to treat this condition before it leads to hypertensive cardiomyopathy. A diagnosis of hypertensive cardiomyopathy encompasses a range of clinical conditions, as assessed by LV changes in geometry, mass, and function. These include concentric remodeling, concentric or eccentric hypertrophy, and filling impairment or early-stage diastolic dysfunction in asymptomatic patients. It is frequently the case that the left atrium (LA) is also affected, resulting in an increase in volume and undergoing geometrical remodeling. In some cases, patients may develop clinical heart failure, presenting with either a preserved or a reduced LVEF. The progression from hypertension to hypertensive cardiomyopathy exhibits considerable variability, contingent on the pressure or volume load and underlying neurohormonal status. However, these differences in LV geometry are likely influenced by a genetic basis. Enhancing comprehension of the mechanisms underlying the development of hypertensive cardiomyopathy in hypertensive patients may facilitate the prevention of cardiovascular events among them [11]. Moreover, systemic hypertension has an independent, but additive effect on aortic stiffness assessed by global pulse wave velocity (PWVg). It is known that PWVg assessed by echocardiography is a marker of CV disease [12].

## 6. Pre-Eclampsia and Its Impact on Cardiac Structure

Pre-eclampsia induces a range of physiological changes that can place significant stress on the heart. Studies have shown that the increased vascular resistance, endothelial dysfunction, and chronic inflammation associated with pre-eclampsia can lead to structural alterations in the myocardium. Specifically, women with a history of pre-eclampsia are at higher risk of developing LVH both during and after pregnancy. A meta-analysis by Melchiorre et al. (2011) found that women who experienced pre-eclampsia showed greater left ventricular mass and wall thickness than those with normotensive pregnancies [13]. These changes reflect the heart’s adaptive response to the increased afterload imposed by the elevated blood pressure common in pre-eclampsia [13].

## 7. Pathophysiology of Left Ventricular Hypertrophy in Pre-Eclampsia

In the pre-eclampsia setting, the development of left ventricular hypertrophy could be the result of an adaptation of LV to chronic increased afterload, and it is intended to be a sign of hypertension-mediated damage [14].

The pathophysiology linking pre-eclampsia to LVH involves several overlapping mechanisms. Endothelial dysfunction is a core component of pre-eclampsia, impairing the ability of blood vessels to relax and increasing vascular resistance, elevating afterload. Additionally, the heightened inflammatory state and oxidative stress characteristic of pre-eclampsia contribute to adverse cardiac remodeling.

Hormonal factors may also play a role, particularly through the renin-angiotensin-aldosterone system (RAAS), which is often dysregulated in pre-eclampsia. This dysregulation can lead to elevated angiotensin II levels, promoting hypertrophic changes in cardiac tissue. Additionally, insulin resistance and dyslipidemia, both common in pre-eclampsia, have been associated with LVH and may exacerbate its development through metabolic and mechanical stress on the heart [15].

## 8. Pre-Eclampsia and Diastolic Dysfunction

Diastolic dysfunction refers to an abnormality in the heart’s ability to relax and fill with blood during diastole, the phase of the cardiac cycle when the heart muscle relaxes [16]. It is a key component of heart failure with preserved ejection fraction (HFpEF) and is often associated with conditions such as hypertension, diabetes, obesity, and aging [17].

The left ventricle plays a central role in diastolic dysfunction. Normally, it relaxes efficiently after contraction, allowing for passive filling with blood from the left atrium. This relaxation is impaired in diastolic dysfunction, leading to increased left ventricular filling pressures [18]. Over time, this can cause symptoms of heart failure, such as shortness of breath, fatigue, and fluid retention, even when systolic function (the heart’s ability to contract) remains normal.

Diastolic dysfunction arises from increased stiffness or reduced compliance of the left ventricle, often due to myocardial fibrosis, hypertrophy, or ischemia. Chronic conditions like hypertension contribute by inducing left ventricular hypertrophy, which impairs relaxation. Additionally, systemic inflammation, endothelial dysfunction, and impaired calcium handling in myocardial cells play significant roles [17].

Echocardiography is the primary diagnostic tool for assessing diastolic function [16,18]. Doppler imaging measures parameters such as mitral inflow patterns and tissue Doppler velocities. Biomarkers like B-type natriuretic peptide (BNP) may also aid diagnosis.

Management focuses on treating underlying conditions. Blood pressure control is essential, particularly with medications such as angiotensin-converting enzyme inhibitors or angiotensin receptor blockers. Lifestyle modifications are also beneficial, including weight loss, exercise, and dietary changes.

Diastolic dysfunction is a progressive condition. Early detection and intervention can help mitigate complications and improve quality of life. Continued research is essential to refine therapeutic strategies for this complex cardiac disorder.

Pre-eclampsia significantly strains the cardiovascular system, leading to structural and functional changes in the heart. Diastolic dysfunction may be related to several factors such as endothelial dysfunction [8], oxidative stress [9], and inflammation, renin-angiotensin-aldosterone system dysregulation [10], impaired calcium handling [19].

Several studies have shown that women with a history of pre-eclampsia are more likely to experience left ventricular remodeling and diastolic dysfunction. Echocardiographic measurements often detect this condition, which shows impaired relaxation and increased left ventricular stiffness [20]. In women with pre-eclampsia, the increased systemic vascular resistance and blood pressure contribute to an increased left ventricular afterload, triggering a remodeling process. This remodeling can lead to increased left ventricular mass and wall thickness, impairing the heart’s relaxation phase and predisposing it to diastolic dysfunction [21]. While some women may experience improvement postpartum, research indicates that others may have persistent diastolic dysfunction, potentially leading to chronic heart issues later in life.

Diastolic dysfunction is a significant predictor of heart failure with preserved ejection fraction (HFpEF), a condition characterized by preserved systolic function but impaired diastolic function. Women with a history of pre-eclampsia are at an increased risk of developing HFpEF, as well as other cardiovascular complications such as ischemic heart disease [22]. Studies suggest that postpartum women with pre-eclampsia should be evaluated for diastolic function, particularly if they exhibit symptoms such as shortness of breath or reduced exercise tolerance.

The American Heart Association recommends that women with a history of pre-eclampsia undergo regular cardiovascular assessments, given the evidence linking this pregnancy complication to long-term cardiovascular health risks [5].

## 9. Atherosclerosis and Pre-Eclampsia

Pre-eclampsia and atherosclerosis are distinct conditions that share similar underlying pathophysiological mechanisms involving endothelial dysfunction, inflammation, and vascular abnormalities. Both conditions have significant implications for maternal and cardiovascular health.

Pre-eclampsia is linked to abnormal placental development, which leads to impaired uteroplacental blood flow, systemic inflammation, and endothelial dysfunction. These vascular abnormalities contribute to hypertension and multi-organ involvement in severe cases.

Atherosclerosis is a chronic inflammatory disease of the arteries caused by the accumulation of lipids, inflammatory cells, and fibrous tissue in the arterial wall. This process leads to plaque formation, arterial narrowing, and reduced blood flow, potentially causing ischemic events like myocardial infarction or stroke. Both conditions exhibit overlapping mechanisms. In pre-eclampsia, there is heightened oxidative stress and impaired nitric oxide production, which mirrors the endothelial dysfunction seen in atherosclerosis. Similarly, inflammatory cytokines and immune activation play pivotal roles in both diseases [23]. The shared pathophysiology has raised awareness of pre-eclampsia as an early marker of cardiovascular risk. Women with a history of pre-eclampsia face a higher lifetime risk of developing atherosclerosis-related cardiovascular diseases, such as coronary artery disease and stroke [24]. This association emphasizes the need for long-term cardiovascular monitoring and preventive care in these women [25]. The association between pre-eclampsia and atherosclerosis underscores the importance of recognizing pregnancy as a window into a woman’s long-term cardiovascular health. Early intervention and post-pregnancy monitoring can reduce the cardiovascular disease burden in women with a history of pre-eclampsia.

## 10. Coronary Disease and Pre-Eclampsia

Younger women with previous preeclampsia had a slightly higher prevalence of coronary atherosclerosis [26]. Indeed, pre-eclampsia can affect epicardial coronary arteries, leading to accelerated atherosclerosis [27], as well as could result in coronary microcirculation dysfunction [28], even if there is not so much evidence in the literature.

The underlying pathophysiological mechanism linking pre-eclampsia with the subsequent development of coronary microcirculation dysfunction is intrinsic to the endothelial dysfunction mechanisms that underpin the development of pre-eclampsia itself: endothelial dysfunction [8,29], angiogenic imbalance [30], oxidative stress [9], and inflammation, renin-angiotensin-aldosterone system dysregulation [10], impaired calcium handling [19]. According to the newest ESC guidelines [31], the concept of chronic coronary syndromes (CCS) includes structural and functional abnormalities in the coronary tree that may cause transient myocardial ischemia. At the microvascular level, coronary microvascular dysfunction (CMD) is increasingly acknowledged as a prevalent factor characterizing the entire spectrum of CCS. Functional and structural microcirculatory abnormalities may cause angina and ischemia even in patients with non-obstructive disease of the large or medium coronary arteries. This is evidenced by the existence of two distinct clinical entities: angina with non-obstructive coronary arteries (ANOCA) and ischemia with non-obstructive coronary arteries (INOCA) [32].

## 11. Relationship Between Pre-Eclampsia and HFpEF

Heart failure with preserved ejection fraction (HFpEF) is a complex syndrome characterized by symptoms of heart failure (e.g., dyspnea, fatigue) despite a normal or near-normal left ventricular ejection fraction (LVEF ≥ 50%) [33]. The pathophysiology of HFpEF is multifactorial, involving abnormalities in the heart’s structure and function as well as systemic and extracardiac processes [34], a complex interplay between cardiac and systemic factors, driven by inflammation, fibrosis, and endothelial dysfunction. Its heterogeneity complicates treatment, as no single pathway explains all aspects of the condition. The hallmark of HFpEF is diastolic dysfunction, defined by impaired LV relaxation and increased stiffness [35]. During diastole, the left ventricle fails to relax adequately, reducing filling and increasing filling pressures; this impairment is often due to myocardial hypertrophy, fibrosis, and altered calcium handling in cardiomyocytes. Myocardial hypertrophy, typically caused by hypertension or aortic stenosis, increases ventricular stiffness, while fibrosis further reduces compliance and contributes to impaired relaxation [17]. Coronary microvascular dysfunction is a prominent feature in HFpEF. Impaired endothelium-dependent vasodilation [36], driven by systemic inflammation and oxidative stress, reduces myocardial blood flow reserve [37], leading to myocardial ischemia, and contributing to stiffening and impaired relaxation of the left ventricle.

HFpEF is closely associated with systemic comorbidities, including hypertension, obesity, diabetes, and chronic kidney disease. These conditions promote a pro-inflammatory state characterized by elevated cytokines and oxidative stress, systemic inflammation triggers endothelial dysfunction and myocardial remodeling. aggravating LV diastolic dysfunction [37]. Obesity, for instance, exacerbates HFpEF through increased plasma volume, systemic inflammation, and altered cardiac metabolism; similarly, diabetes contributes by inducing advanced glycation end-product (AGE) accumulation, oxidative stress, and microvascular dysfunction.

HFpEF involves abnormal interaction between the left ventricle and arterial system [38]. Increased arterial stiffness, common in aging and hypertension, raises afterload and worsens LV diastolic function, the inability of the left ventricle to compensate for arterial stiffness results in elevated systemic vascular resistance and pulmonary congestion. Increased left atrial pressures, secondary to impaired LV filling [16,17,18,35], contribute to pulmonary venous congestion and pulmonary hypertension (PH). Over time, PH can lead to right ventricular (RV) dysfunction, compounding heart failure symptoms. RV dysfunction worsens systemic congestion, further aggravating the clinical picture of HFpEF. The left atrium plays a critical role in maintaining adequate ventricular filling by acting as a reservoir and pump [35]. In HFpEF, atrial remodeling and dysfunction due to elevated filling pressures impair this function. Atrial fibrillation, common in HFpEF, further reduces diastolic filling and exacerbates symptoms. At the cellular level, HFpEF involves impaired calcium cycling, mitochondrial dysfunction, and altered sarcomeric protein function. These changes reduce cardiomyocyte relaxation efficiency and contribute to increased stiffness [17,35].

Several kinds of literature evidence demonstrate a prognostic relationship between pre-eclampsia and Heart Failure (HF) [39,40,41,42], particularly with HFpEF [43]. In a report from the New York and Florida Healthcare Cost and Utilization Project (N = 2,532,515), the adjusted hazard ratio for hospitalization for heart failure with preserved ejection fraction (HFpEF) in patients with a history of pre-eclampsia was 2.1 [44]. Unlike heart failure with reduced ejection fraction (HFrEF), pathophysiological mechanisms that determine an increased risk of developing heart failure with preserved ejection fraction (HFpEF) in patients with a previous history of pre-eclampsia include left ventricular hypertrophy, diastolic dysfunction, and the possible development of myocardial fibrosis [45,46]. Hypertensive disorders of pregnancy (HDP) have been linked to subclinical alterations in cardiac function. The precise mechanism underlying this phenomenon remains unclear; however, elevated levels of soluble anti-angiogenic proteins, such as soluble fms-like tyrosine kinase-1 (sFlt1), may play a role. Elevated circulating levels of anti-angiogenic proteins in HDP correlate with and may contribute to myocardial dysfunction as measured by the Global Longitudinal Strain Ultrasonographic Technique [47]. Other manifestations of diastolic dysfunction, a main pathophysiological aspect of HFpEF, in HDP, include impaired myocardial relaxation, an increase in left ventricle filling pressures, and increases in LV mass index (LVMI) [47].

At one year postpartum, approximately 45% of women with early-onset [48,49] and severe pre-eclampsia [50] demonstrate grade I–II diastolic dysfunction. The incidence of worsening diastolic function is reported within two years postpartum, with a higher prevalence observed in women with early-onset pre-eclampsia. This dysfunction may persist for at least 10 years postpartum [51].

## 12. Pre-Eclampsia and Peripartum Cardiomyopathy

Pre-eclampsia and peripartum cardiomyopathy (PPCM) are significant complications of pregnancy, both of which involve cardiovascular dysfunction and can lead to serious maternal and neonatal morbidity and mortality. While they are distinct conditions, they share overlapping features and risk factors, and pre-eclampsia itself is a well-recognized risk factor for PPCM [52]. A meta-analysis revealed that the prevalence of pre-eclampsia in women with peripartum cardiomyopathy is 22%, which is more than four times the estimated global average [53]. A registry of the EURObservational Research Program, including 411 women with peripartum cardiomyopathy, revealed that 22.8% had experienced pre-eclampsia [54]. PPCM is a rare form of heart failure that occurs during the last month of pregnancy or within five months postpartum. It is characterized by left ventricular systolic dysfunction, typically with an ejection fraction of <45%, in the absence of another identifiable cause. Symptoms include fatigue, dyspnea, orthopnea, and peripheral edema, resembling other forms of heart failure [55]. Pre-eclampsia is a significant risk factor for PPCM. Both conditions share systemic endothelial dysfunction as a common pathophysiological feature, such as prolactin fragmentation by trophoblastic matrix metalloproteinases and cardiac angiogenic imbalance [56,57,58]. Pre-eclampsia may act as a trigger for PPCM in genetically or metabolically predisposed women, suggesting that the two conditions may represent a spectrum of cardiovascular disorders associated with pregnancy. Women with pre-eclampsia often experience increased vascular and oxidative stress, both of which predispose them to myocardial injury; furthermore, elevated salt-1 levels in pre-eclampsia may exacerbate the anti-angiogenic and cardiotoxic environment in PPCM [47,52]. sFLT1 is a soluble form of fms-like tyrosine kinase 1, a substance with anti-angiogenic and probably cardiotoxic effects, which may be of significance. sFLT1 is secreted by the placenta as pregnancy progresses and also in the perinatal period. Subclinical dysfunction of cardiomyocytes may result from impaired mechanisms that protect the heart against anti-angiogenic factors or from increased secretion of this substance, which is observed in PPCM. Its concentration is also increased in pre-eclampsia in the early stages of pregnancy, even before a diagnosis is made [56,59,60]. Furthermore, it has been observed that an angiogenic imbalance, characterized by an elevated sFLT1/PLGF ratio, can precipitate the development of heart failure [61]. It has been postulated in the literature that another substance with anti-angiogenic effects, sVEGFR1 (soluble version of vascular endothelial growth factor receptor-1), which disrupts homeostasis in various vascular beds, may also play a role in the pathogenesis of the aforementioned diseases [62,63].

## 13. Pre-Eclampsia and Postmenopausal Cardiovascular Risk

Pre-eclampsia is increasingly recognized not merely as a pregnancy-specific disorder, but as a cardiometabolic stress test that unmasks a woman’s predisposition to chronic diseases later in life—particularly cardiovascular disease (CVD).

Women with a history of pre-eclampsia have a 2- to 4-fold increased risk of developing chronic hypertension, ischemic heart disease, stroke, heart failure, and peripheral arterial disease.

This elevated risk may become apparent as early as 5 to 10 years postpartum and can persist for decades. Moreover, pre-eclampsia is associated with markers of subclinical cardiovascular damage, including atherosclerosis, increased arterial stiffness, and coronary artery calcification—even in the absence of clinically overt disease [64].

Menopause represents a critical transition in a woman’s cardiovascular trajectory. The natural decline in estrogen levels is linked to increased arterial stiffness, elevated blood pressure, unfavorable shifts in lipid profiles, greater visceral adiposity, insulin resistance, and the onset of metabolic syndrome [65].

In women with a history of pre-eclampsia, these menopause-associated changes may amplify pre-existing vascular injury, accelerating the development and severity of CVD. Thus, menopause should be viewed not only as a hormonal milestone but also as a period of heightened cardiovascular vulnerability in this at-risk population [64].

## 14. Clinical Implications

Understanding the connection between pre-eclampsia and cardiovascular disease (CVD) presents a crucial opportunity to identify and address cardiovascular risks early in high-risk populations. Implementing regular postpartum cardiovascular evaluations, particularly for women who have experienced severe or recurrent pre-eclampsia, can play a pivotal role in preventive healthcare strategies. Healthcare providers should incorporate a patient’s pre-eclampsia history into their cardiovascular risk assessments and offer personalized guidance on adopting heart-healthy lifestyle practices, such as maintaining a balanced diet, engaging in regular physical activity, and quitting smoking. These proactive measures have the potential to significantly reduce the risk of developing CVD in the future.

To ensure the effectiveness of cardiovascular prevention strategies in women with a history of hypertensive disorders of pregnancy (HDP), it is essential to improve postpartum screening methodologies.

A promising approach involves the development of an HDP-specific, female-centered cardiovascular risk stratification model. As a first step, routine cardiovascular assessments should be implemented for women with a history of pre-eclampsia, with particular attention to blood pressure, lipid profiles, and fasting plasma glucose levels.

Several clinical strategies have been identified to reduce long-term cardiovascular risk in this population. Lifestyle interventions are of primary importance.

A randomized controlled trial demonstrated that a six-month postpartum program combining nutritional counseling with structured cardiovascular exercise significantly reduced arterial stiffness in women with a history of severe HDP. Notably, participants attained vascular health metrics comparable to those of women without prior complications [66,67,68].

Moreover, early modification of health behaviors, including dietary improvements, has been shown to positively influence cardiovascular risk factors following HDP. Nevertheless, many affected women do not meet national dietary guidelines, highlighting the need for tailored nutritional interventions [69].

In addition to lifestyle modifications, pharmacological strategies may also play a dual role—not only in mitigating cardiovascular risk in women with a history of pre-eclampsia, but potentially in preventing the onset of pre-eclampsia itself. Pre-eclampsia must be recognized, based on scientific evidence, as a condition with long-term health implications extending beyond the peripartum period.

Low-dose aspirin (75–100 mg/day) is widely accepted in current clinical practice for primary cardiovascular prevention, and accumulating evidence suggests that it also lowers the risk of pre-eclampsia when initiated early in high-risk pregnancies [70,71].

Additionally, a strong association exists between elevated LDL-cholesterol, cardiovascular risk, and obesity—with obesity itself being a well-established risk factor for pre-eclampsia. Consequently, lifestyle and dietary interventions targeting weight management and lipid control in women with prior pre-eclampsia are crucial components of preventive care.

However, when diet alone is insufficient to manage dyslipidemia, pharmacological lipid-lowering therapies may be beneficial. Preclinical studies in murine models have shown that pravastatin improves outcomes related to pre-eclampsia [72], and further research has demonstrated that it can reduce circulating levels of soluble fms-like tyrosine kinase-1 (sFlt-1), a key molecule implicated in the pathophysiology of pre-eclampsia [73,74].

Thus, the appropriate use of lipid-lowering agents in women with elevated LDL-cholesterol may not only reduce their long-term cardiovascular risk but also potentially influence disease-modifying pathways linked to pre-eclampsia.

## 15. Future Implications

In view of the aforementioned points, it would be advisable for doctors and patients to approach pre-eclampsia as a condition with the potential to have a permanent impact on patients’ lives and to significantly influence long-term cardiovascular risk. The initial step in this process is to undertake a basic evaluation of the risk factors for pre-eclampsia, including arterial hypertension, obesity, and diabetes mellitus.

Utilizing the tools provided by the European Society of Cardiology guidelines on cardiovascular prevention, it is then necessary to identify patients at the highest cardiovascular risk. This enables the planning of targeted preventive strategies, which are based on the promotion of a healthy lifestyle and on appropriate intervention, including pharmacological treatment, for all those risk factors that are common to both pre-eclampsia and cardiovascular diseases.

Indeed, the preventative role of low-dose acetylsalicylic acid in cardiovascular diseases is well established, as is the role of lipid-lowering therapy in this context. It is hypothesized that these same pharmacological agents could also play a role in the prevention of pre-eclampsia itself.

## 16. Future Research Directions

Future research into the relationship between pre-eclampsia and long-term cardiovascular risk is increasingly focusing on genetic and biological mechanisms, in order to better understand disease progression and to predict future outcomes. Key directions include genomic and epigenetic studies that aim to clarify how genetic variants contribute to both pre-eclampsia and cardiovascular disease [75].

Epigenetic changes, such as alterations in DNA methylation patterns during or following pre-eclampsia, may predispose women to future cardiovascular dysfunction [76]. It would also be important to identify biomarkers—such as soluble fms-like tyrosine kinase-1 (sFlt-1) and placental growth factor (PlGF)—that persist in the postpartum period and could help stratify long-term cardiovascular risk.

Moreover, the investigation of molecular pathways—including endothelial dysfunction, oxidative stress, and systemic inflammation—may reveal novel therapeutic targets for early intervention. Furthermore, factors leading to vascular remodeling and persistent endothelial alterations after delivery are key areas of study and could play an important role in the stratification of long-term cardiovascular risk.

In the current era of precision medicine, each of these research domains could, in the future, contribute to the development of a personalized model of cardiovascular risk stratification in patients who have experienced pre-eclampsia.

## 17. Conclusions

The connection between pre-eclampsia and long-term cardiovascular risk underscores the importance of recognizing this pregnancy complication as a critical predictor of future health outcomes. Research highlights the need to raise awareness among healthcare providers and patients, emphasizing the value of proactive monitoring and lifestyle modifications. Further investigation into the genetic and biological mechanisms underlying this relationship could pave the way for targeted interventions to mitigate cardiovascular risk in women with a history of pre-eclampsia, potentially offering health benefits to their offspring as well.
